# Unleashing the full potential of digital outcome measures in clinical trials: eight questions that need attention

**DOI:** 10.1186/s12916-024-03590-x

**Published:** 2024-09-27

**Authors:** Mia S. Tackney, James R. Carpenter, Sofía S. Villar

**Affiliations:** 1 MRC-Biostatistics Unit, University of Cambridge, East Forvie Building, Forvie, Robinson Way, Cambridge, CB2 0SR UK; 2 MRC Clinical Trials Unit at University College London, Institute of Clinical Trials and Methodology, 90 High Holborn, London, WC1V 6LJ UK; 3 Department of Medical Statistics, London School of Hygiene and Tropical Medicine, Keppel St, London, WC1E 7HT UK

**Keywords:** Digital health technology, Digital outcome measures, Validation, Clinical trial, Digital endpoints

## Abstract

The use of digital health technologies to measure outcomes in clinical trials opens new opportunities as well as methodological challenges. Digital outcome measures may provide more sensitive and higher-frequency measurements but pose vital statistical challenges around how such outcomes should be defined and validated and how trials incorporating digital outcome measures should be designed and analysed. This article presents eight methodological questions, exploring issues such as the length of measurement period, choice of summary statistic and definition and handling of missing data as well as the potential for new estimands and new analyses to leverage the time series data from digital devices. The impact of key issues highlighted by the eight questions on a primary analysis of a trial are illustrated through a simulation study based on the 2019 Bellerophon INOPulse trial which had time spent in MVPA as a digital outcome measure. These eight questions present broad areas where methodological guidance is needed to enable wider uptake of digital outcome measures in trials.

**Supplementary Information:**

The online version contains supplementary material available at 10.1186/s12916-024-03590-x.

## Background

### Potential and opportunities for digital outcome measures

In clinical trials, patients’ health outcomes are measured to assess whether a new intervention is safe and effective. Digital health technologies (DHTs), such as wearable devices, sensors, implantables and software applications (apps), offer new ways of measuring outcomes in clinical trials. Examples of digital outcome measures include the following: passive collection of physical activity data through accelerometers [1, 2], passive monitoring of sleep duration through smartwatches [3], active data collection such as digital versions of walk tests via a smartwatch and associated smartphone app [4], and virtual motor examinations conducted via smartwatches [5]. We note that DHTs also offer opportunities for digital interventions in trials (e.g. digital behaviour change interventions [6]), but this is outside the scope of this article.

Digital outcome measures offer several potential benefits to patients’ experiences of trials. Firstly, they allow outcomes which typically require hospital visits, such as the 6 Minute Walk Test or polysomnography, to be measured more frequently and in patients’ chosen locations. This reduces burden and cost for patients and their carers to travel to the clinic and enables trial participation to be more inclusive for people who are geographically distant or whose mobility is restricted [7]. Such burdens are particularly exacerbated in rare diseases where patients may need to travel longer distances to a clinic or research centre [8]. Secondly, outcomes captured in patients’ daily contexts may be more meaningful and better reflect patient experiences compared to in-clinic assessments which are prone to bias due to factors such as fatigue or motivation [9]. Thirdly, digital outcome measures may widen inclusion criteria due to their potential to measure a wider range of physiological measurements. For example, trials which use the 6 Minute Walk Test typically exclude patients who score below a threshold at baseline, since they are expected to be non-ambulant at the time of follow-up [10]. In contrast, using digitally measured physical activity outcomes can potentially allow inclusion of such individuals in trials [11].

From a statistical perspective, DHTs offer opportunities for improved outcome measures but also pose new challenges. In terms of advantages, digital outcome measures may be more accurate and sensitive than traditional outcome measures which require diaries or questionnaires and are prone to recall and social desirability bias [12, 13]. For primary outcomes, shifting to a more sensitive digital approach can have important implications for sample size calculations [14]. For example, Servais et al. (2021) estimate that the change of outcome measure from the 6 Minute Walk Test to the digital Stride Velocity 95th Centile (SV95C) would reduce the number of patients required to detect stabilisation of symptoms in Duchenne muscular dystrophy from 100 to 30 patients per arm [11]. Digital outcome measures enable high-frequency measurements in patients’ daily conditions, which can reduce the high variability of episodic “snapshot” assessments in clinic [15, 16]. However, important challenges include: measurements being taken in an uncontrolled setting where contextual factors may be unknown; the requirement for patients to engage with digital devices without medical supervision, which can lead to complex patterns in missing data [17]; and new post-randomisation events caused by, for example, technical problems or changes in software [18].

### Current landscape

Despite an increase in the use of digital technology for clinical trials, the uptake of digital outcome measures is limited. For example, in neurology trials, the percentage of trials using DHTs registered in ClinicalTrials.gov increased from 0.7% in 2010 to 11.4% in 2020 but was mostly used for testing functionality or clinical usability of the DHT or as digital interventions (for example in apps to increase medication adherence) [19]. A systematic review of trials in PubMed, CENTRAL, and EMBASE with digital outcome measures identified 75 trials, of which 57% used a physical activity related digital outcome measure, 24% of which tested a pharmacological intervention and 47% of which used a DHT to measure a primary outcome [7].

Digital outcome measures require regulatory endorsement if they are used as primary or secondary outcomes in a phase IIB or phase III trial to support an efficacy claim for a new intervention. Validation can be pursued under the Food and Drug Administration (FDA) either as a digital biomarker (a marker of a biological process, such as heart rate) or a clinical outcome assessment (COA) (an assessment of how a patient feels, functions or survives, such as a patient-reported outcome) [20]. Izmailova et al. (2023) discuss the potentially subtle distinction between biomarkers and COA for digital outcome measures [21]. Qualification can also be pursued by the European Medicines Agency (EMA) [22]. Once qualified, the outcome measure is considered sufficiently reliable to have a specific interpretation in drug development and regulatory review.

There are only a few examples of regulatory approved digital outcome measures, and at the time of writing, no medical product has yet been approved using a digital outcome measure [23]. Specifically, we are aware of three digital outcome measures that have received regulatory endorsement. Firstly, in 2018, the EMA adopted the patient-reported outcome, PROactive, which combines patient experiences with physical activity data from DHTs for trials in chronic obstructive pulmonary disease [24]. Secondly, the EMA qualified the digital outcome measure *Stride Velocity 95 Centile (SV95C)* as a secondary outcome for trials in Duchenne muscular dystrophy in 2019 and subsequently as a primary outcome in 2023 [25, 26]. Thirdly, in 2023, the FDA agreed to Bellerophon’s proposal to use time spent in moderate-to-vigorous physical activity (MVPA) as a primary outcome in a phase III trial in fibrotic interstitial lung disease [27]. While this does not equate to gaining qualification status of a digital outcome measure in the sense that SV95C has achieved, it serves as the first digital outcome measure where the FDA agreed for its use as a primary outcome in a specific study. We provide further details on the latter two examples in the “Case study: SV95C for Duchenne muscular dystrophy” and “Case study: time spent in MVPA for pulmonary hypertension associated with fibrotic interstitial lung disease” sections. The limited number of regulatory approved digital outcome measures presents a major roadblock for their uptake [23].

### Methodological challenges

There is a growing body of literature and guidance around digital outcome measures from regulatory authorities [20, 22, 28] and multi-stakeholder consortia [15, 29]. In addition to the evidence dossier for the regulatory-approved SV95C [11, 25, 26], there are examples of study proposals for rheumatoid arthritis [30] and a proposal for an evidence dossier for validation of outcome measures from mobile sensors [31]. However, there is a lack of methodological guidance for validation as well as deployment of digital outcome measures in clinical trials. Some areas which require further guidance were highlighted in a comment to the FDA’s recent draft guidelines on Digital Health Technologies for Remote Data Acquisition in Clinical Investigations [32]. The lack of guidance coupled with the limited number of exemplars are roadblocks [23] which may make investigators overly reliant on the few existing exemplars this may lead to the use of examples such as SV95C as templates for validation without sufficient consideration of the issues that new contexts raise.

**Fig. 1:**
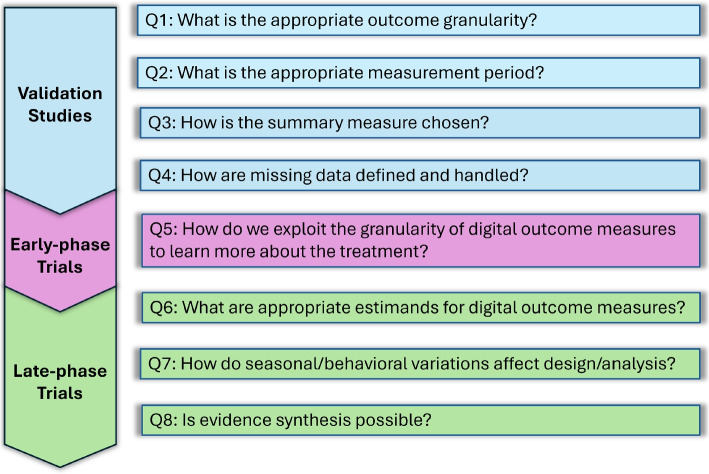
Eight questions for digital outcome measures: Q1–Q4 relate to validation, Q5 relates to early phase trials, and Q5–Q8 relate to late phase trials

In this article, we provide an overview of methodological aspects that need attention in order to fully integrate digital outcome measures into clinical trials practice. After providing a background in the “Validation” section, we outline the “Questions for validation of digital outcome measures”. These four questions are illustrated through SV95C for Duchenne muscular dystrophy as a case study. We then outline the “Questions for deploying digital outcome measures in trials”. One of the four questions presented here is more relevant to early-phase trials, while the other three questions are more relevant to late-phase trials. These questions are illustrated through the 2019 phase II Bellerophon trial as a case study, which used time spent in MVPA as a digital outcome measure. Figure 1 provides a roadmap of the presented questions. In the “Simulation study: key questions illustrated in a primary analysis” section, we illustrate the practical importance of issues highlighted by these questions in a primary analysis through a simulation based on the on 2019 Bellerophon study on INOPulse. In the “Discussion”, we review the ground we have covered and put into context the importance of the eight questions in accelerating development and deployment of digital outcome measures.

## Validation

**Fig. 2:**
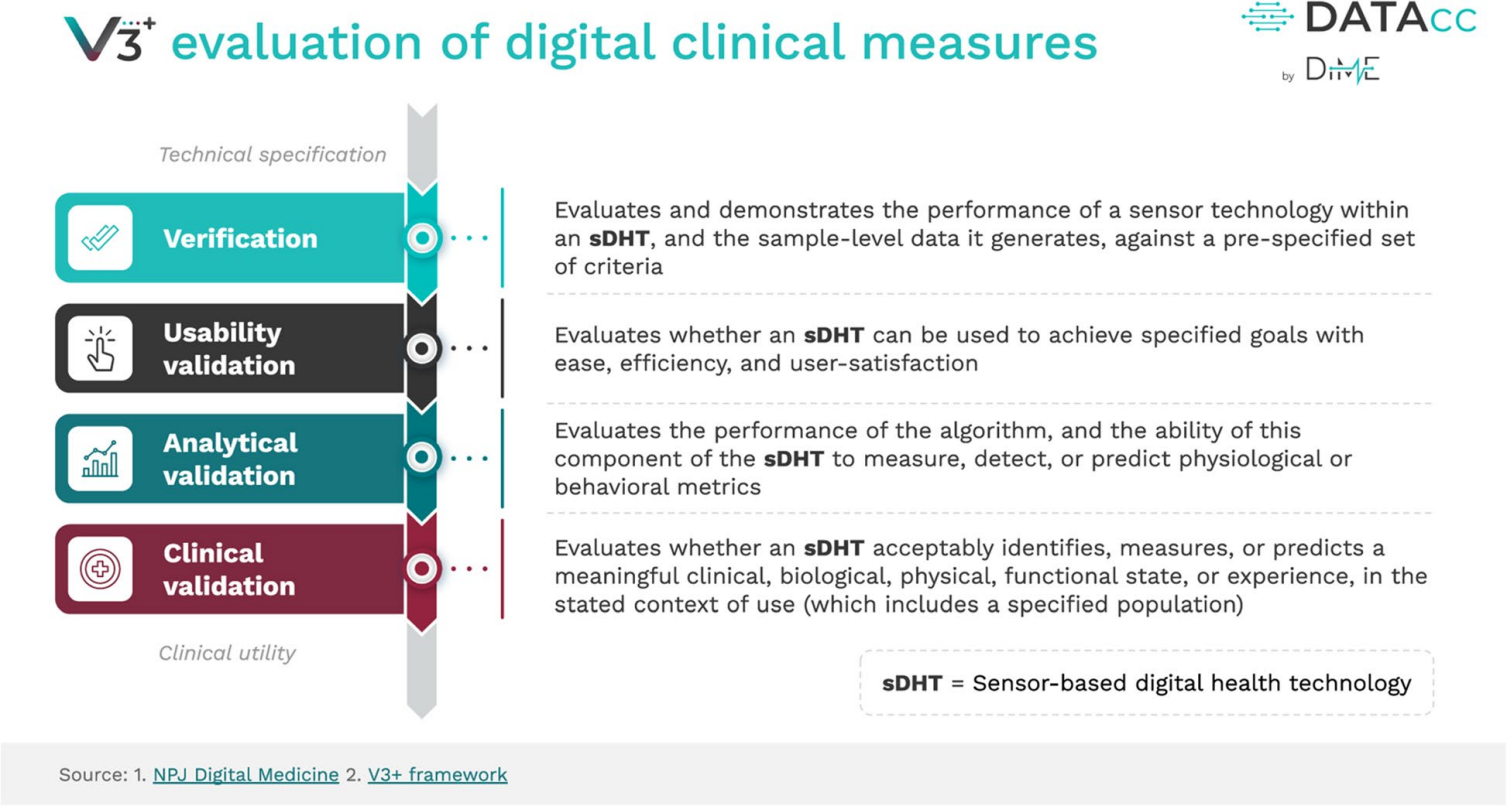
V3+ framework from the Digital Medicine Society. Original source: [33], reprinted with permission

Novel digital outcome measures need to be validated before they can be used in a clinical trial. We describe validation via the recently pioneered V3+ framework [29, 33], illustrated in Fig. 2, which decomposes validation into several aspects: verification, user validation, analytical validation and clinical validation. We describe each of these aspects, using SV95C as an illustrative example, before posing our four methodological questions for validation.

### Case study: SV95C for Duchenne muscular dystrophy

Duchenne muscular dystrophy is a rare, genetic neuromuscular disease which affects young children [34], and gold-standard outcomes are in-clinic exercise capacity tests such as the 6 Minute Walk Test. A digital outcome measure for physical activity was developed using an ankle-worn DHT developed by ActiMayo and a later-generation version by Syde. After a 10-year journey, Stride Velocity 95th Centile (SV95C), resulting from 180 hours of wearing the device, was approved by the EMA as a secondary outcome in 2019 [25] for patients who are 5 years of age and above in pivotal or exploratory drug therapeutic studies. It was later approved as a primary outcome in 2023 [26]. It is undergoing approval by the FDA through the Clinical Outcome Assessment programme [8], and work is ongoing to allow use of SV95C for other progressive neuromuscular diseases [26].

### Conceptualisation of a digital outcome measure

A novel outcome measure should reflect a meaningful aspect of health (MAH), regardless of whether it is captured digitally. These are aspects of a health condition that patients may want to improve, want to prevent, or want not to worsen []. The outcome should capture a specific and measurable aspect of the MAH, which is defined as the concept of interest (COI) [35]. The novel outcome should have a clear context of use (COU), which is a description of how the new outcome should be used and in what contexts and populations [28, 29].

### Verification

Verification is the evaluation of the performance of sensors within devices. The sensors should be tested against pre-specified criteria in bench-top tests by hardware manufacturers, prior to any testing on human subjects.

### Usability validation

Usability validation evaluates whether the DHT can achieve “specified goals with ease, efficiency and user-satisfaction” [33]. This could involve, for example, participatory observation of patients interacting with the device in an actual or simulated environment, interviews or focus groups.

Usability can have important methodological implications. For example, devices that are more comfortable will lead to better compliance and more complete data collection [35, 36]. The frequency of data collection can affect the granularity of the data obtained and the variability of the summaries over a given measurement period. In the SV95C case study, the Actimayo/Syde device collected data at 100 Hz, considering a balance between more precise outcomes as a result of higher frequency and longer battery life which can affect usability of the device.

### Analytical validation

Analytical validation evaluates how well the digital outcome measure reflects the physiological/behavioural outcome that one intends to capture and can include demonstration of accuracy, repeatability and robustness to a range of physiological and environmental factors. In Table 1, we set out properties that comprise analytical validation, the statistical approach taken in SV95C and details of the approach.

**Table 1:** Aspects of analytical validation for SV95C for Duchenne muscular dystrophy

Property	Statistical approach taken in SV95C	Details
**Accuracy**	Compute mean difference between the digital and traditional outcome measures and its standard deviation.	Distance walked in 6 min measured by the wearable device was compared to the distance measured by physiotherapists. The difference was 0.75 m ± 8.9. Stride speed measured by the wearable was compared to that measured by a motion capture room (wearable device detected 98.7% of the strides).
**Repeatability**	Calculate test-retest reliability using the intra-cluster correlation (ICC), which measures relatedness of repeated responses.	ICC between individuals’ first 15 days and second 15 days within a 1-month baseline recording were computed. SV95C had a high ICC of 0.937.
**Robustness**	Plot variability of digital outcome measure vs length of measurement period and identify when stability is observed. Compare variability for environmental/contextual factors.	Low variability of 4.41% was observed for SV95C after 180 h of wear time. No differences were found between morning and afternoon, but significant differences were found between weekdays and weekends.

### Clinical validation

Clinical validation evaluates whether the DHT data measures a meaningful clinical state or experience in the context of use and whether a specific clinical question can be answered with the digital outcome measure. Clinical validation includes demonstrating whether the digital outcome measure accurately measures the COI through evidencing correlation with established outcomes and showing whether it can discriminate between a group with a disease condition and a healthy control group. Furthermore, sensitivity to change over time is typically assessed, either in terms of change due to disease progression or positive change due to treatment through a longitudinal study. In Table 2, we set out properties that comprise clinical validation, the statistical approach taken in SV95C and details of the approach.

**Table 2:** Aspects of clinical validation for SV95C for Duchenne muscular dystrophy. 6MWD: 6 Minute Walk Distance, NSAA: North Star Ambulatory Assessment, 4SC: 4 Stair Climb

Property	Statistical approach taken in SV95c	Details
**Known-groups validity**	Compare median of digital outcome measure between patients with disease and healthy controls.	Median of SV95C in DMD population (1.583 m/s) was lower than that of age-matched healthy controls (2.713 m/s).
**Convergent validity**	Compute correlations between digital and traditional outcome measures.	Moderate correlations between SV95C and traditional outcomes were observed: 6MWD (0.542) , NSAA (0.645) and 4SC (0.547) at baseline. Similar results were observed at 3, 6, 9 and 12 month follow-ups.
**Sensitivity to disease progression**	Compute change in median of digital outcome measure between baseline and follow-up. Compare with traditional outcome measures.	A decline from baseline was established at 3 months for patients on a stable regimen of corticosteroids or having initiated corticosteroids from at least 6 months. SV95C was demonstrated to be more sensitive to disease progression than traditional outcome measures.
**Sensitivity to change due to treatment**	Calculate change in median of digital outcome measure in patients who have started on a treatment.	A positive change in median was observed for patients who were started on corticosteroids. Patients in two clinical trials investigating new medicinal interventions were followed up, but the interventions were shown not to be effective.

## Questions for validation of digital outcome measures

We have set out the key components of validation through the V3+ framework. We now present four methodological questions related to validation which have received limited attention. We continue to use SV95C as a case study to illustrate these questions in a specific example.

### Q1: What is the appropriate outcome granularity?

DHT data can be extracted and aggregated at different levels of granularity. For example, the raw signal can be aggregated at the epoch-level (e.g. minute-level), or it could be aggregated at a coarser day- or week-level. See Appendix 2 for an illustration of the hierarchical structure of DHT data. The possibility of defining outcomes at different granularities opens several new considerations.

Firstly, different components of validity may require data to be aggregated at different granularities. For SV95C, accuracy was assessed by evaluating stride speed and distance walked in 6 min, whereas repeatability and robustness were evaluated at a much coarser level of the SV95C summary statistic (see Table 1). There is a need to clarify the granularity of data needed for different aspects of validation. Secondly, aggregation typically requires decision-making on restrictions such as removal of outliers or definition and handling of missing data, which introduces areas of discretion. There is therefore a need to provide transparent documentation (e.g. via an open source data processing pipeline) clarifying how outcomes at different granularities are obtained. Thirdly, some processing and aggregation may be done by proprietary software of a DHT, and decisions on restrictions, for example, may be unknown to the investigator. This limits the reproducibility of the study if changes are made to the software or a slightly different DHT is used. To resolve this, there is encouragement to use DHTs that provide raw data and to use open source software to convert raw signals into epoch-level summaries [15].

### Q2: What is the appropriate measurement period?

Validation studies require participants to wear or engage with a DHT for a period of time. Complex considerations factor into the decision of how long the measurement period should be. For example, a period of 180 hours (which can be achieved in 15 days with 12 hours of recording a day) was chosen as sufficient for establishing a baseline or follow-up SV95C measurement due to the following reasons [25]:

- 180 h is considered long enough to reduce the variability from day-to-day activities, such as differences between weekdays and weekends;
- As the trial population is children, it may be important to consider measurement periods that are longer than two weeks as children of separated parents may experience different home environments from one week to the next;
- The measurement period should not be so long as to cause burden on the participant;
- The measurement period should not be so long that disease progression could happen over the course of this period;
- Compliance declines when the measurement period is made longer; 90% of patients recorded 180 hours over a period of one month, but only 79% of patients were compliant over a longer period of 6 months.

We note a number of other factors may need to be considered in other settings, including learning effects and observer effects. When DHTs use data from active tasks, for example in digital cognitive assessments or digital walk tests, there may be a learning effect during the initial attempts as the participant becomes used to the device/software. The initial attempts to complete a task in an app may be regarded as a familiarisation phase [37, 38]. Furthermore, for DHTs that monitor participants’ daily activities, the observer effect or Hawthorne effect may have an influence on outcomes, as the awareness of being observed can typically modify participants’ behaviour. Participants whose physical activity is being monitored may increase their activity early on in the trial, when their sense of being observed is at its peak. These are important considerations for selecting a measurement period, and in some cases, it may be suitable to discard an initial period in a familiarisation phase. Thus, the selection of the length of measurement period requires awareness of several factors, which are not limited to those outlined in the SV95C evidence dossier. Implications of the selection of the measurement period on a primary analysis are explored in the simulation study in the “Impact of observer effect (Q7) and measurement period (Q2)” section.

### Q3: How is the ideal summary measure chosen?

The choice of summary measure to obtain a single value from the measurement period is another complex decision. For Duchenne muscular dystrophy, several candidate mobility-related variables were selected, including stride length (median and 95th centile), stride velocity (median and 95th centile) and total distance walked per hour. Amongst these selected five outcomes, stride length and velocity were significantly correlated to the gold-standard exercise capacity tests, and SV95C was the most sensitive to change due to deterioration of the underlying disease. Furthermore, sensitivity due to positive change as a result of steroid treatment was demonstrated for SV95C [26], leading to the selection of SV95C as the selected digital primary/secondary outcome [25].

There are several open questions in how such a decision should be made in other Contexts of Use. For example, why were the 50th and 95th centiles chosen, and not other centiles or other summary statistics? If there are several candidate measures which perform to different extents across the validation metrics, it is unclear how the importance of different components of validation should be weighted in the selection of a single summary measure. Furthermore, if several candidate measures perform equally well across the validation metrics, how should one select a single summary statistic? SV95C was chosen as it was more sensitive to change, but it is not clear how one single metric should be selected in other contexts. Furthermore, sensitivity to change may not be possible to demonstrate in disease areas where no effective treatment exists. Finally, there are also questions around how the chioce of granularity (Q1) and measurement period (Q2) can affect validation performance of a chosen summary measure. These are several methodological areas where the choices made for SV95C cannot easily be translated into other contexts.

### Q4: How are missing data defined and handled?

As digital outcome measures typically require data collection for a longer period of time and without medical supervision, data are likely to be missing for a number of reasons. These include technical problems with the device or decline in participants’ engagement with the study. DHT data can be aggregated at different levels (e.g. epoch-level, day-level, week-level), which makes defining missing data a difficult task. Most studies use a threshold on wear-time to define a missing observation. For SV95C, only participants with at least 50 hours of recording were considered complete observations, and for longitudinal analyses, patients who were followed for at least over 3 months and had at least 50 hours in each recording period [26] were considered complete observations. Izmailova et al. (2018) note heterogeneity in definitions of missing data in digital outcome measures, which is a potential barrier to evidence synthesis [12]. We discuss this further in the “Q8: Will evidence synthesis be possible?” section.

There is a need to align estimands, assumptions for the missing data and analysis approaches. Analyses conducted in the evidence dossier for SV95C implicitly assume that data are missing completely at random (MCAR), since only complete observations were included. If data were not MCAR, there is a risk that the summary statistics obtained are biased. For example, it is likely in general that individuals in the study may choose to remove the device on days when they feel unwell and are less mobile. In this scenario, the estimated SV95C would be biased upwards. Methods for handling missing accelerometer data using multiple imputation (MI) under different missing data mechanisms have been proposed [39, 40]. Furthermore, Di et al. (2022) provide a review of missing data methods for digital outcome measures related to physical activity and blood glucose [17]. The implications of making incorrect assumptions on the missing data mechanisms on the results of a primary analysis of a clinical trial are explored in the simulation study in the “Impact of Missing Data (Q4)” section.

## Questions for deploying digital outcome measures in trials

Having set out questions for the validation of digital outcome measures, we now pose four questions for the deployment of digital outcome measures in clinical trials. The transition from traditional to digital outcomes opens new challenges in the design and analysis of trials. One out of the four questions relate to early-phase trials, and the remaining three questions relate to late-phase trials. In this section, we illustrate the questions through the 2019 Bellerophon study on INOPulse which used time spent in MVPA as a digital outcome.

### Case study: time spent in MVPA for pulmonary hypertension associated with fibrotic interstitial lung disease

The 2019 Bellerophon trial was a phase II trial which aimed to assess whether inhaled nitric oxide improved physical activity in patients with pulmonary hypertension associated with interstitial lung disease (see Appendix 1 for more details). The trial had two exploratory digital outcome measures: average time spent in moderate-to-vigorous physical activity (MVPA) and total activity, which were both measured by an Actigraph wrist-worn accelerometer [27] in a 1-month measurement period. One of the objectives of the phase II trial was to identify which digital outcome measure should be used in a pivotal phase III trial. Since the phase II trial demonstrated differences between treatment and control groups for average time spent in MVPA, this outcome was approved for use as a primary outcome in a phase III trial. Using a digital outcome measure instead of a questionnaire-based outcome allowed the sample size to reduce from 300 to 140 in the phase III trial [41]; however, there was no improvement for the treatment group over control in average time spent in MVPA and the trial was stopped for futility [42].

### Q5: Can we exploit the granularity of digital outcome measures to learn more about the treatment?

While the primary analysis of a trial with a digital outcome measure will typically focus on a single summary measure (e.g. SV95C or average time spent in MVPA), the granular time series data can be leveraged to learn more about the treatment, which may be relevant particularly for early-phase trials. For example, the granular data can provide an understanding of circadian patterns and how these are impacted by treatment. Such questions call for statistical methods beyond those typically used in the trials setting. Lisi and Abellan (2023) analyse epoch-level actigraphy data using generalised additive models (GAMs) [43] which include a sum of parametric and non-parametric smooth terms in the linear predictor [44]. The smooth terms allow circadian patterns in activity to be characterised and differences in these patterns can be compared by covariates, treatment group or timepoint. They demonstrate that, in one study on amyotrophic lateral sclerosis (ALS) patients, physical activity at baseline peaks in the middle of the day, but by week 48 has a marked dip in activity in the middle of the day [43].

Several approaches to analysis of time series DHT data in observational settings may be beneficial in the analysis of trials. For example, Functional Data Analysis, which models data using functions or functional parameters, has been used to model the 24-hour physical activity profile as an outcome in a regression [45–47]. Using a Functional Data Analysis framework, global effects of covariates or treatment on the diurnal outcome can be tested as well as local tests for the effect for a given part of the day. For DHTs that passively monitor patients over a long period of time (implantables, for example), methods such as changepoint detection have the potential to provide insight into the timing when patients may respond to treatment and can also help to characterise the nature of the change, such as an abrupt shift or a slow improvement [48]. These insights may help characterise the mechanism of action of a treatment and could provide more precise information into the time that it takes for patients to respond to treatment (compared to snapshot in-clinic measurements). Changepoint detection could also help define futility rules in the context of adaptive trial designs by specifying the time at which an ineffective treatment can be dropped as no response to treatment is being observed.

### Q6: What are appropriate estimands for digital outcome measures?

Estimands clarify the scientific question being investigated in a study; informally, the estimand captures the treatment effect we wish to estimate and the population we wish to estimate it for [49]. Thus, the estimands framework is a structured description of a trial’s estimand to facilitate alignment between the trial aims, the study design and planned analysis. The five attributes of an estimand [50] are:

- Population of interest for the intervention;
- Variable used to address the trial objective (also known as endpoint);
- Handling of post-randomisation events (also known as intercurrent events);
- Treatment or intervention;
- Population level summary.

Izem et al. (2024) describe how the estimands framework can help trialists systematically think through ways in which decentralisation can impact each attribute of the estimand [18]. Trialists may consider decentralizing several different aspects of a trial, such as recruitment, delivery of treatment, data collection and monitoring. They illustrate that considering the impact on the estimand can clarify the added value, potential risks and potential for novel estimands that decentralisation can offer. Here, we illustrate how the estimands framework can help identify the impact of selecting a digital outcome (as opposed to a traditional outcome) in terms of the attributes of the estimand.

In the Bellerophon INOpulse study, actigraphy outcome measures were used, offering a novel and patient-centred *Variable*. Actigraphy reflects changes in real-world daily physical activities that are relevant to patients [51], so the use of the digital outcome measure may better reflect a scientific question centred around patient experiences. We note that the Bellerophon INOpulse study required both actigraphy and the in-clinic 6WMT, but studies that replace the 6MWT with actigraphy outcome measures may allow improved alignment to the target *Population* of interest as they may enable inclusion of participants in geographically remote areas [52]. On the other hand, there may be complications such as the exclusion of individuals who are unable or unwilling to use digital devices. Digital outcome measures may open potential for new *Post-randomisation Events*, such as technical issues with the device or updates in software for the digital device [22]. In this specific example, the 6MWT requires patients to be ambulatory, so an injury or hospitalisation may be potential post-randomisation events for a traditional outcome measure which may not necessarily be post-randomisation events for a digital outcome measure. Furthermore, connected devices may offer opportunities for improved monitoring of post-randomisation events such as treatment discontinuation. Table 3 summarises the impacts that one may consider when shifting from a traditional to a digital outcome measure, in a setting similar to the Phase II Bellerophon trial on INOPulse.

**Table 3:** Possible impact of shifting from a traditional to a digital outcome measure for a set-up similar to the phase II Bellerophon trial for INOPulse

	Impact of outcome measure on estimand attribute	
Estimand Attribute	6MWT (traditional)	Time spent in MVPA (digital)
Variable: physical activity	Easily affected by fatigue/motivation and has high variability. One-time snapshot of patients’ health.	Affected by seasonal/behavioural factors. May be a better reflection of patients’ daily experiences.
Population: patients with fibrotic interstitial lung disease, ages 18 to 85 years, need for supplemental oxygen by nasal cannula	Potential exclusion of individuals in more remote locations or those unable to attend in-clinic assessments.	Potential exclusion of patients who are unwilling or unable to use digital devices.
Post-randomisation events	Requires patients to be ambulatory; therefore, injuries or hospitalisation are potential post-randomisation events	Technical issues with devices or updates in software may introduce new post-randomisation events.
Treatment: inhaled nitric oxide (iNO) or placebo delivered via INOpulse	No impact.	No impact
Population level summary	A single outcome is generated per test in a controlled environment, so the choice of population level summary is relatively straight forward.	Choice of population level summary is more complex. Day- and month-level summaries and definitions of missing data on each level are needed. Seasonal/behavioural variations may need to be considered.

### Q7: How do seasonal/behavioural variations in digital outcome measures affect the design and analysis of trials?

A clinical trial to assess a new intervention typically includes a baseline measurement period before the intervention is introduced, and a follow-up measurement period after the intervention. The timings of these measurement periods require careful consideration. Digital outcome measures related to daily physical activity, for example, may be affected by season as individuals tend to be more active in warmer months and more sedentary in colder months [53] and will be more prone to seasonal variation compared to outcome measures captured in controlled clinical settings.

In the Bellerophon INOpulse study, there was a 3-month gap between baseline and follow-up measurement periods, and recruitment occurred between January to July. Patients recruited in the winter months (e.g. January and February) may have a follow-up period where they are more active than baseline due to a seasonal effect, in addition to any changes due to the treatment. Thus, individual-level differences between baseline and follow-up may be affected by seasonal variation. Due to randomisation, the seasonal effect generally does not incur bias in the estimate of the treatment effect; this highlights the importance of concurrent controls. However, standard error of the treatment effect can be inflated, and in the specific case where there is an interaction between the treatment and season, for example if an exercise intervention is particularly effective in the summer, the estimated effect of treatment will be biased. There may also be variations in digital outcome measures induced by behavioural aspects. For example, individuals in a physical activity study may be more active in an initial period due to the observer or Hawthorne effect. The impact of such seasonal/behavioural effects on the primary analysis is explored in the simulation study in the “Impact of seasonal effect (Q7)” section.

Additionally, an important observation is that the length of measurement period for the Bellerophon INOpulse needed reassessment. Initially, participants’ baseline physical activity was assessed during a 1-week measurement period, but it was noted that “the actigraphy data during run-in was insufficient to provide an appropriate baseline. Given this, the first month of data immediately following randomisation was used to allow an adequate period of time to accurately determine baseline data for both groups” [27]. The reason why the data during the one-week run-in was insufficient is unclear, but it highlights that a measurement period which appears sufficient for the purposes of validation may later appear to be insufficient in the context of a clinical trial.

### Q8: Will evidence synthesis be possible?

The need for standardised digital outcome measures has been noted in the literature [12, 31]. Systematic reviews have noted the hetereogeneity of digital outcome measures [54]. For example, Graña Possamai et al (2020) reported 266 digital outcomes from 21 diabetes trials [7]. The heterogeneity in outcomes make evidence synthesis through methods such as meta analysis very challenging. Furthermore, current reporting standards for digital outcome measures may be insufficient to make evidence synthesis possible. Graña Possamai et al. (2020) note a lack of reporting on the validity and reliability of the DHT used and on how missing data are defined and handled [7].

Recent developments to help researchers identify suitable digital outcome measures include a Library of Digital Endpoints by the Digital Medicines Society (DiMe), which is continually updated with digital outcome measures used in industry-sponsored studies [55]. More broadly, there are several initiatives for improving standardisation and reporting of trials [56], but some additional guidance may be needed to address challenges specific to digital outcome measures. For example, the Core Outcome Set Standard for Development (COS-STAD) provides an agreed standard for the set of outcomes that should be measured and reported, as a minimum, in trials for a specific area of health [57, 58]. Core Outcome Sets are defined through a consensus process. DHTs may be a viable way of measuring Core Outcomes, but the increased levels of discretion for digital outcome measures may make it more challenging to achieve consensus.

## Simulation study: key questions illustrated in a primary analysis

Through a simulation study, we illustrate the importance of carefully considering some of the questions described in this paper. Specifically, we illustrate the potential impact for the primary analysis of a trial when these key questions are overlooked. We report the aims, data-generating mechanism, estimand, method, and performance measures (ADEMP) of the simulation study, following the structure introduced by Morris et al. (2019) [59].

### Aim

Through a set-up that closely mimics the analysis of average time spent in MVPA in the 2019 Bellerophon phase II study, this simulation study aims to illustrate the impact of these questions on the primary analysis:

- How do seasonal/behavioural variations in digital outcome measures affect the design and analysis of trials? (Q7)
- What is the appropriate measurement period? (Q2)
- How are missing data defined and handled? (Q4)

We illustrate the impact on the mean and standard error the estimated effect of treatment.

### Simulation set-up

#### Data generating mechanism

This simulation generates data similar to the 2019 Bellerophon phase II study (see Appendix 1 for more details). In each repetition, we generate data on daily time spent in MVPA over 28 days at follow-up for 44 participants. Participants are randomised to treatment or control with a 2:1 ratio. Simulation results when participants are randomised with a 1:1 ratio are shown in the Supplementary File. We denote by $y_{ij}$ the daily time spent in MVPA at follow-up for patient *i* on day *j*, for $i \in \left\{ 1, 2, ..., 44 \right\}$ and $j \in \left\{ 1, 2, ..., 28 \right\} .$ We assume that $y_{ij}$ has a log-normal distribution with mean $\mu _{ij}$ and standard deviation 46. We model $\mu _{ij}$ as:

1 $$\begin{aligned} \mu _{ij} & = \text {baseline}_i+ \delta _1 I(\text {arm}_i=1)\nonumber \\ & + \delta _2 I( \text {patient}\ i\ \text {recruited in winter})\nonumber \\ & + \delta _3 I( \text {patient}\ i\ \text {recruited in winter}) I(\text {arm}_i=1)\nonumber \\ & + \delta _4 I(\text {day}\ j\ \text {in week 1}) +\delta _5 I(\text {day}\ j\ \text {in week 1}) I(\text {arm}_i=1)\nonumber \\ & + \alpha _i \end{aligned}$$

where

- $\texttt {baseline}_i$ is the average of the daily time spent in MVPA during the baseline period of 1 month, which we assume has a log normal distribution with mean 77 and standard deviation 52. The log normal distribution was selected as average daily time spent in MVPA is known to be right-skewed [60], and the values of the mean and standard deviation were selected to be similar to those observed in the trial [27].
- $\delta _1$ is the treatment effect and is assumed to be 12.5, a value similar in magnitude to the effect observed in the trial [27]. For ease of interpretation, the simulation is set up such that the experimental group experiences an increase in MVPA, while the placebo group experiences no change. In the Belleorphon INOPulse study, the experimental group maintains their baseline activity levels while the placebo group experiences a deterioration.
- $\delta _2$ is the additive effect of season if an individual is recruited in the winter and have a follow-up in the summer (and therefore whose outcome is influenced by the season).
- $\delta _3$ is the effect due to an interaction between season and treatment.
- $\delta _4$ is the additive effect of an observer effect, which leads to increased physical activity in the first week.
- $\delta _5$ is the effect due to an interaction between the observer effect and treatment.
- $\alpha _i \sim N(\mu =0, \sigma ^2=4)$ is a random effect for participant *i*.

The simulation study explores how the following scenarios can lead to different implications for the results of a primary analysis:

- Seasonality (Q7): the added effect $\delta _2$ in Eq. (1) is set to vary between 0 and 10. The proportion of the cohort who are recruited in winter (and therefore whose outcome measure is influenced by season) is set to 0.1, 0.2 or 0.5. We consider scenarios with an interaction between seasonal effect and treatment (where $\delta _3=0.1 \delta _2$) and without an interaction (where $\delta _3=0$).
- Observer effect (Q7) and measurement period (Q2): the observer effect in week 1, $\delta _4$, is set to vary between 0 and 10. The impact of this observer effect may depend on the length of the measurement period. Therefore, two measurement periods are considered: 2 weeks and 4 weeks. We consider scenarios with an interaction between observer effect and treatment (where $\delta _5=0.1 \delta _4$) and without an interaction (where $\delta _5=0$).
- Missing data (Q4): the proportion of participants who have missing data are set to be 0.1, 0.2 or 0.5, and the proportion of missing days for those participants are set to vary between 0 and 0.5. Two missing data mechanisms are considered: (1) days are missing completely at random (MCAR), where for each participant who has missing data, a proportion *p* of randomly selected days are set to missing (the missingness indicator is unrelated to any observed or unobserved variable), and (2) days are missing not at random (MNAR): where for each participant who has missing data, a proportion *p* of days with the lowest physical activity levels are set to missing.

We note that the model for the daily time spent in MVPA is kept simple to highlight the impact of a few key decisions made in the design and analysis of trials that use digital outcome measures. Considerations for a more realistic model are provided in Appendix 3.

#### Estimand

We denote by $\bar{y}_{i,.}$ the average of the compliant days during the follow-up period:

2 $$\begin{aligned} \bar{y}_{i,.} = \sum \limits _{j=1}^{28} y_{ij} I(\text {participant}\ i\ \text {is compliant on day}\ j), \end{aligned}$$

noting that a compliant day in the Bellerophon INOPulse study is defined as a day with at least 600 minutes (10 hours) of wear time while awake. Furthermore, a month-long measurement period is compliant (and therefore included in the analysis) if there are at least 14 compliant days.

We assume that the primary analysis of the trial is:

3 $$\begin{aligned} \bar{y}_{i,.} = \beta _0 + \beta _1 \text {baseline}_i + \beta _2 \text {treat}_i + \epsilon _i, \end{aligned}$$

where we assume $\epsilon _i \sim N(0, \sigma ^2)$.

The actual primary analysis included baseline stratification factors as fixed effects, which we omit here for simplicity [27].

The estimand of interest is the estimated treatment effect ($\beta _2$ in Eq. (3)) and its standard error.

#### Methods

This simulation study considers only one method of estimating the treatment effect: Eq. (3), since the aim is to illustrate scenarios where this method may lead to misleading conclusions.

#### Performance measure

The performance measures in this study are the mean of the treatment effect across and the mean of the standard error of the treatment effect across 10,000 repetitions. Simulations are run in the R Statistical Software (version 4.3.1) [61].

### Results

#### Impact of seasonal effect (Q7)

**Fig. 3:**
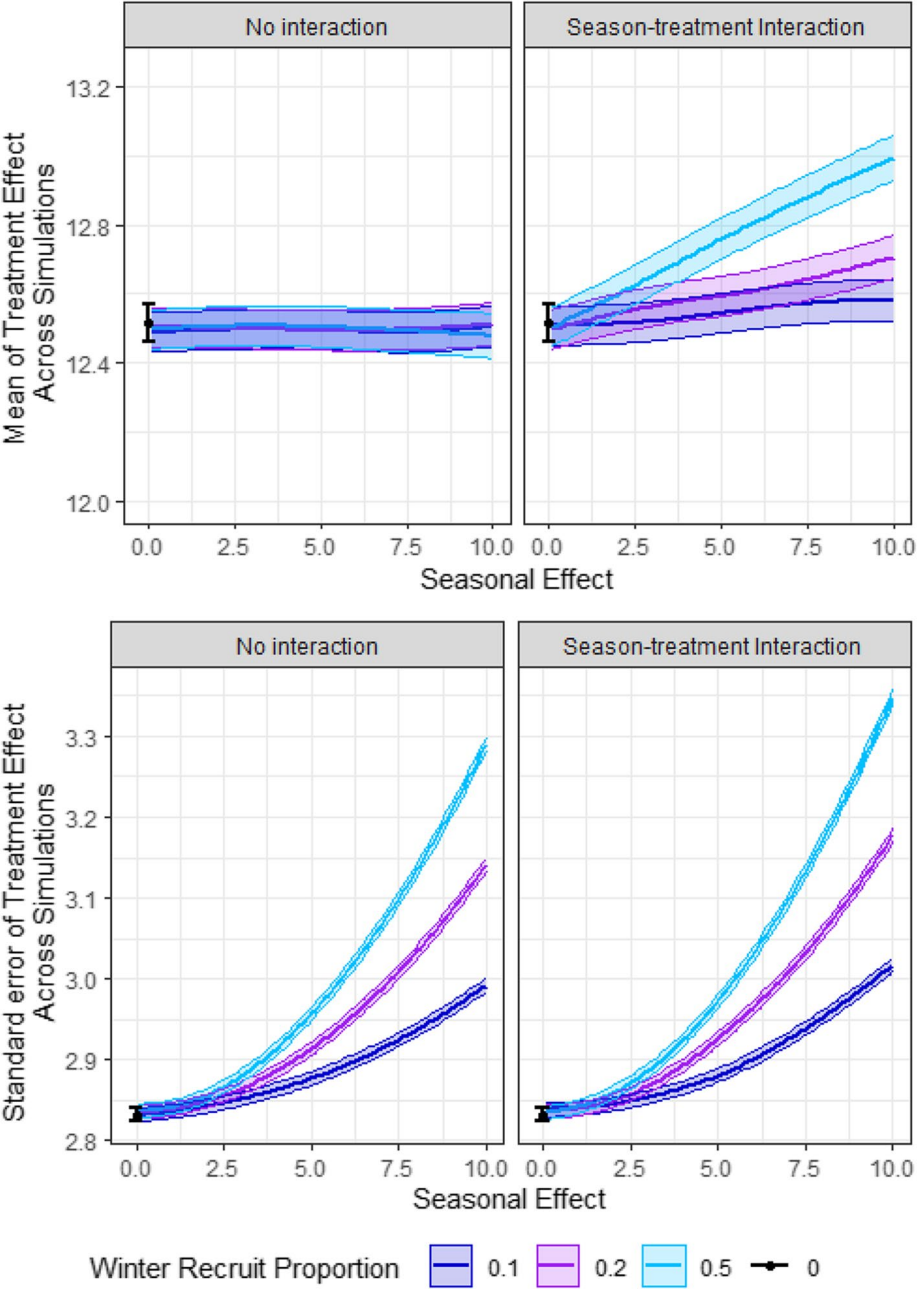
Seasonality. Plots show estimated mean of treatment effect (top) and its standard error (bottom) without interaction between season and treatment (left) and with an interaction (right). The seasonal effect varies between 0 and 10. Error bars indicate $1.96 \times$ Monte Carlo error. The black error bars indicates the scenario under no seasonal effect. Dark blue, purple and teal lines indicate that the proportion of patients recruited in winter are 0.1, 0.2 and 0.5, respectively. Results are based on 10,000 simulations

In Fig. 3, we display the mean of the treatment effect (top panels) and the standard error of the treatment effect (bottom panels) without an interaction between season and treatment (left) and with an interaction (right). Error bars indicate $1.96 \times$ Monte Carlo error. The black error bars indicate results where there is no effect of season. We observe that seasonality does not incur bias in the treatment effect when there is no interaction, due to randomisation of the treatment. Seasonality leads to increased standard errors, particularly in the case when a larger proportion of the study experiences a seasonality effect. This increase in standard error will lead to reduced power to detect the effect of treatment. In the presence of an interaction between treatment and season, we observe bias in the treatment effect, which increases with the proportion of participants who experience seasonal variation as well as with the strength of the seasonal effect. While a treatment such as INOPulse may be unlikely to interact with seasonal effect, we note that exercise interventions, for example, may be likely to interact with season.

#### Impact of observer effect (Q7) and measurement period (Q2)

**Fig. 4:**
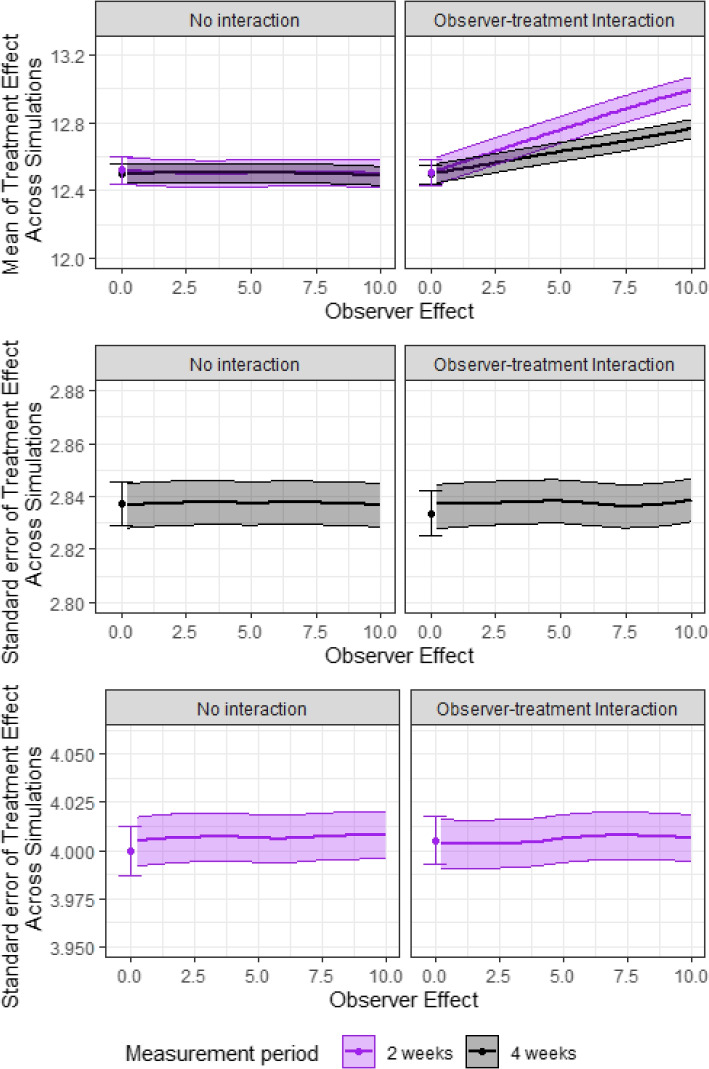
Observer effect and measurement period. Plots show the estimated mean of treatment effect (top), standard error when the measurement period is 4 weeks (middle) and standard error when the measurement period is 2 weeks (bottom), without an interaction between the observer effect and treatment (left) and with an interaction (right). Note that the scale of the *y*-axis is different for the middle and bottom panels. The observer effect varies between 0 and 10. Error bars indicate $1.96 \times$ Monte Carlo error. Grey lines indicate when the measurement period is four weeks, and purple lines indicate when the measurement period is 2 weeks. Results are based on 10,000 simulations

In Fig. 4, we display the mean of the treatment effect (top panels), standard error of the treatment effect when measurement period is four weeks (middle panels) and standard error of the treatment effect when measurement period is 2 weeks (bottom panels) without an interaction between the observer effect and treatment (left) and with an interaction (right). Error bars display $1.96 \times$ Monte Carlo error. Grey lines indicate when the measurement period is 4 weeks, and purple lines indicate when the measurement period is 2 weeks. We observe that the standard error is increased substantially when the measurement period is reduced from 4 weeks to 2 weeks. Similarly to seasonality, this increase in standard error will lead to reduced power to detect the effect of treatment. Due to randomisation of the treatment, the observer effect does not lead to bias in estimated effect of treatment in the absence of an interaction between treatment and observer effect. However, if there was an interaction between treatment and the observer effect, we observe bias.

#### Impact of missing data (Q4)

**Fig. 5:**
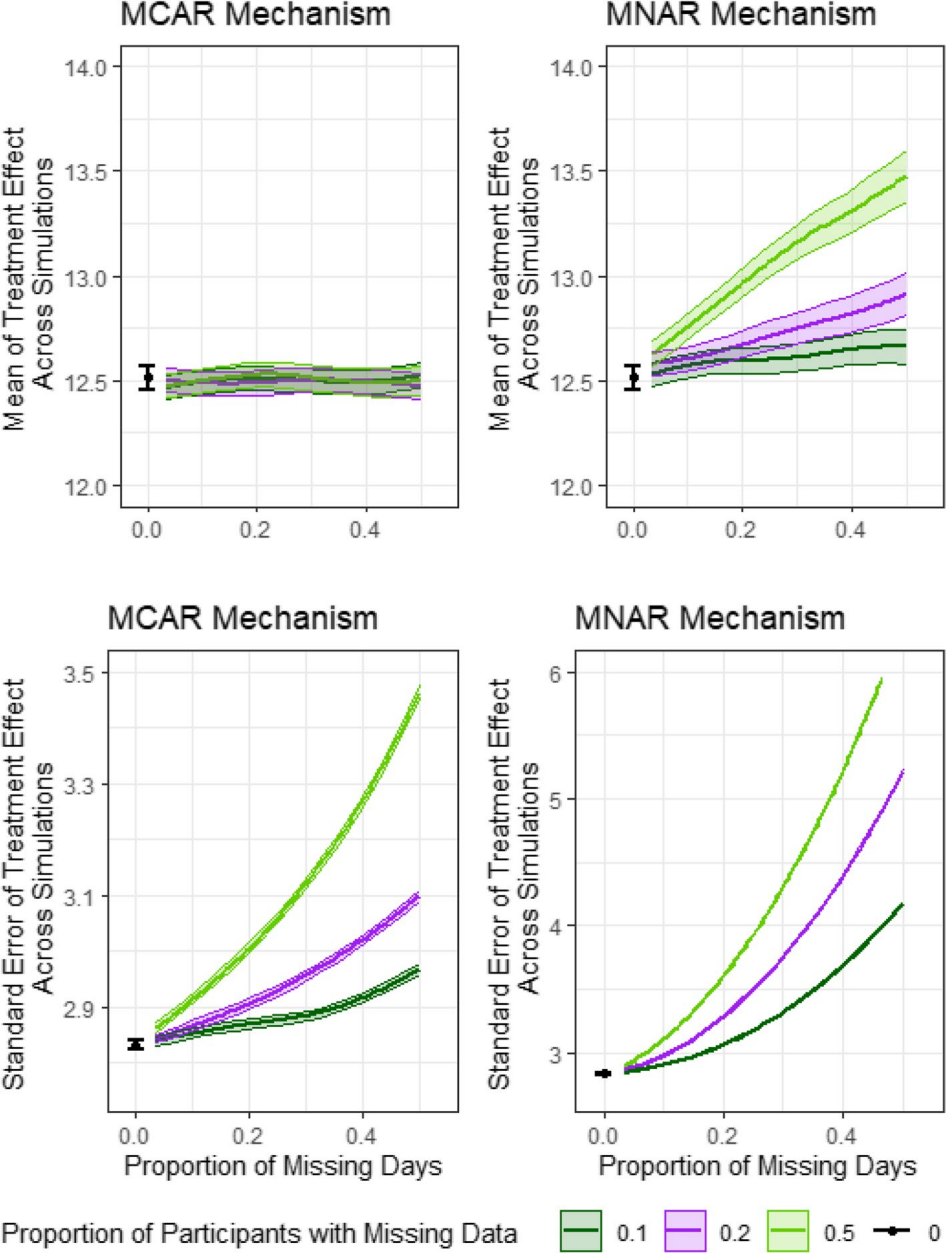
Missing data. Plots show the change in the estimated mean of treatment effect (top) and its standard error (bottom) when data are MCAR (left) and MNAR (right). The proportion of days that are missing completely at random varies between 0.05 and 0.5. Error bars indicate $1.96 \times$ Monte Carlo error. The black error bar indicates the scenario under complete data. Dark green, purple and light green lines indicate that the proportion of patients with missing data are 0.1, 0.2 and 0.5, respectively. Note that the scale of the *y*-axis is different for the left and right panels for standard error. Results are based on 10,000 simulations

In Fig. 5, we display the mean of the treatment effect (top) and the standard error of the treatment effect (bottom) under an MCAR mechanism (left) an under and MNAR mechanism (right). Under the MCAR mechanism, we do not observe systematic bias in the effect of treatment; however, we observe increases in standard error as the proportion of missing days, as well as the proportion of participants with missing data, increases. Similarly to the other simulation scenarios, the increase in standard error will affect the power to detect an effect of treatment. Under an MNAR mechanism, there is an upward bias in the estimate of the treatment effect as the extent of missing data increases as well as a dramatic increase in the standard error. The potential for bias is potentially large when data are MNAR, highlighting the need to assess robustness to missing data assumptions.

The Supplementary File provides simulation results where participants are randomised to treatment or control with a 1:1 ratio. Similar conclusions are drawn for the scenarios that lead to bias and increases in standard error, and changes to the magnitude of the standard error are observed.

## Discussion

There are several methodological aspects that need consideration to fully integrate digital outcome measures in clinical trials. The V3+ framework provides a unified terminology for validation, but guidance is needed on appropriate choices on the granularity of the outcome, length of measurement period, choice of summary measure and handling of missing data in new contexts. Methodological research to provide clarity in these areas is particularly pertinent, given that the regulatory process of digital outcome qualification is a lengthy and expensive process: the qualification of SV95C as a secondary outcome took over 10 years. Methodological guidance will facilitate acceleration of of the validation process.

Going beyond validation and into the deployment of digital outcome measures in trials, there are additional methodological opportunities and challenges. For early-phase trials, the high-frequency digital outcome measures can help answer new scientific questions about the mechanism of action of a treatment. Guidance is needed on statistical methodology to exploit the granularity of the outcome, such as flexible approaches to modelling via functional data analysis, or time series methods to identify changes in physiology due to treatment. For late-phase trials, new challenges arise around appropriate estimands and handling seasonal and behavioural variations. Clarity in these methodological aspects and good practices in reporting are needed for evidence synthesis to be possible in the future.

The simulation study demonstrated potential consequences of overlooking these methodological challenges in the primary analysis of a trial with digital outcome measures. Firstly, if there is seasonal variation in the digital outcome measure, the standard error of the treatment effect may increase, impacting power to detect the effect of treatment. If there is an interaction between the seasonal effect and treatment, there will be bias as well as an increase in standard error. Similar impacts on the treatment effect are seen if there is risk of an observer effect. The simulation study illustrated that the choice of measurement period can have a large impact on the standard error, which can affect the power of the study. Finally, when the digital outcome measure is MCAR, the standard error of treatment effect will increase, and if the digital outcome measure is MNAR, there will be bias as well as an increase in the standard error.

While there are some design and analysis strategies to mitigate consequences illustrated in the simulation study, further methodological work and guidance is needed. Approaches to reduce the impact of seasonality at the design stage include recruiting at appropriate times of the year and stratifying for season in the randomisation scheme [62]. Furthermore, treatment imbalance within seasons could exacerbate the impact of a potential seasonal effect. Careful implementation of modern randomisation techniques, such as maximum tolerated imbalance (MTI) procedures, which limit treatment imbalances per season and at the end of the study, could help to mitigate this issue [63]. In the analysis of the trial, adjusting for season and potentially the season-treatment interaction can help mitigate the consequences on standard error and bias. To mitigate the impact of an observer or learner effect, there may be a need to consider discarding an initial part of the measurement period. Finally, there is a need to for guidance on sensitivity analyses to missing data assumptions, as digital outcome measures are likely, in part, to be MNAR, but standard approaches to handling missing data typically assume that data are MCAR or MAR. Robustness to plausible departures from the MAR assumption has been explored for accelerometer outcomes [17, 39], but guidance for a wider range of digital outcome measures is needed.

We note that there are additional methodological challenges which have not been explored in this paper. These include the following: clinical validation of novel digital outcome measures for aspects of a disease or concept of interest for which there is no gold standard [23]; clinical validation in disease areas where no disease-modifying therapies of symptomatic treatments exist, as demonstrating sensitivity to treatment cannot be demonstrated; and establishing a minimally important clinical difference (MCID) for novel digital outcome measures. A practical challenge is the limited number of validated medical devices to measure digital outcome measures, which restrict limit investigators’ choices. While the Clinical Trials Transformation Initiative (CTTI) recommends that investigators should first identify the desired characteristics of a device and then select an appropriate DHT that fits these requirements [15], in practice, investigators may need to change the choice of outcome measure or trial design so that it fits the capabilities of the DHT.

We posed eight methodological questions which provide a comprehensive view of areas which need attention to accelerate the validation and deployment of digital outcome measures. Through addressing these questions, the full potential of digital outcome measures can be unleashed.

## Supplementary Information

Supplementary Material 1.

## Figures and Tables

**Fig. 6:**
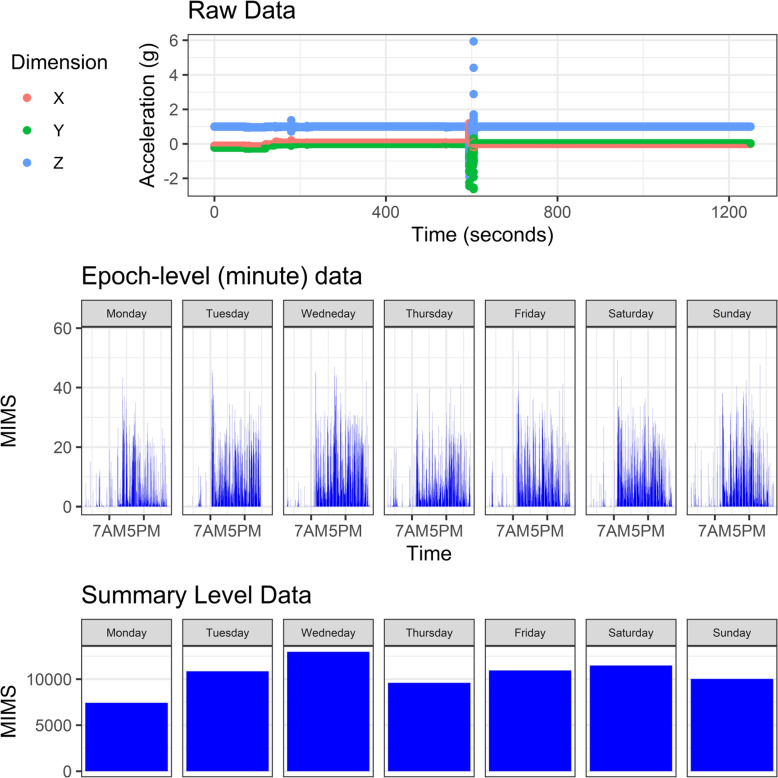
Daily summaries of MIMS triaxial are displayed for one participant. The mean MIMS triaxial value for the week is 10480.66

## Data Availability

Not applicable.

## Appendix 1

The phase II Bellerophon trial had three objectives relating to inhaled nitric oxide (iNO) via INOpulse as a treatment for patients with fibrotic interstitial lung disease interstitial lung disease:

1. To establish the safety and efficacy of a higher dose of iNO $(45 \mu \text {g/kg IBW/h})$ than what was tested in a pilot study $(30 \mu \text {g/kg IBW/h})$;
2. To assess whether the benefit observed in the pilot study (over 2 months) would be sustained in a longer period (4 months);
3. To assess a number of exploratory outcome measures in order to identify optimal outcome measures to include in a phase 3 study.

Expanding on the third aim, the outcome measures that were explored included:

- 6 Minute Walk Test (6MWT)
- Accelerometer outcomes: time spent in moderate-to-vigorous physical activity (MVPA, min/day), overall activity (step counts/day);
- Patient reported outcomes: University of California San Diego shortness of breath questionnaire (UCSD SOBQ); St. George’s Respiratory Questionnaire (SGRQ).

Of 44 patients randomised between January and July 2019, 30 patients were on treatment and 14 on placebo. A summary of the results of the trial are that:

- The 6 Minute Walk Test did not lead to a significant difference between treatment and control groups;
- Accelerometer outcomes: treatment group maintained their physical activity while placebo group experienced a decline (placebo-corrected difference in MVPA of 12.3min/d, approximately $16\%$). In overall activity, there was no significant difference.
- Patient reported outcomes: treatment group on average reported stable symptoms while placebo group reported worsening assessment on UCSD SOBQ. This result was clinically meaningful but not statistically significant. Similar results were observed in the SGRQ.

## Appendix 2

Data collected from DHTs can be classified in a hierarchical structure of data types, based on the level of processing and aggregation of the raw signal [17]:

1. **Raw data**
  Raw data refers to data extracted directly from a DHT. For example, the top panel of Fig. 6, raw data are shown from an accelerometer in the 2013–2014 National Health and Nutrition Examination Survey (NHANES) [64]. Acceleration in the unit of g is measured in the *x*-, *y*-, and *z*-directions at every 1/80th of a second (80 Hz). The plot displays $10^5$ datapoints per dimension, which amounts to 1250 seconds of data. Device manufacturers vary on whether they make the raw signal data accessible to users [15].
2. **Epoch-level data**
  Raw sensor data are processed into a health-related outcome and summarised at the epoch-level. An epoch is the smallest time granularity for which the summary measure can be provided. For example, in the middle panel in Fig. 6, minute-level Monitor Independent Movement Summary Unit (MIMS) for 7 days for one participant is is displayed, which is the epoch-level data for the NHANES accelerometer dataset. In many settings, these summaries are typically produced by the device's proprietary software. There is encouragement to use open source software to convert raw signals into epoch-level summaries instead (if raw signals are available) [15].
3. **Summary data**
  The epoch-level data are typically aggregated to form summary measures on a coarser level. The bottom panel in Fig. 6 shows the total MIMS value for each day for the same participant. An even coarser summary is the average of daily step counts across the week (10480.66 steps). These summaries are usually used in the primary analysis of a clinical trial.

## Appendix 3

A simplified model for time spent in MVPA is provided for the simulation study in Eq. (1). A more realistic model for daily time spent in MVPA may:

- Use a zero-inflated distribution, such as a zero-inflated Poisson or zero-inflated negative binomial distribution, as there will likely be a proportion of days where participants do not engage in MVPA [60].
- Use a right-truncated distribution, as daily time spent in MVPA will likely have an upper limit specific to the population. The increased physical activity due to treatment, seasonal effect or observer effect would be limited by this upper bound.
- Be autocorrelated.

## Abbreviations

4SC: 4 Stair Climb
6MWD: 6 Minute Walk Distance
6MWT: 6 Minute Walk Test
ALS: Amyotrophic lateral sclerosis
COI: Concept of interest
COU: Context of use
CTTI: Clinical Trials Transformation Initiative
EMA: European Medicines Agency
DHT: Digital health technology
DMD: Duchenne muscular dystrophy
FDA: Food and Drug Administration
GAM: Generalised additive model
ICC: Intra-cluster correlation
MAH: Meaningful aspect of health
MCAR: Missing completely at random
MCID: Minimally clinically important difference
MAR: Missing at random
MNAR: Missing not at random
MVPA: Moderate-to-vigorous physical activity
NSAA: North Star Ambulatory Assessment
SV95C: Stride Velocity 95th Centile

## Data availability

No datasets were generated or analysed during the current study.

## Code availability

R code is available on GitHub: https://github.com/mst1g15/DHT_simulation.
