# Supplementary material for: Unleashing the full potential of digital outcome measures in clinical trials: eight questions that need attention
Source: BMC Med. 2024 Sep 27;22:413. doi: 10.1186/s12916-024-03590-x (PMC11438362; doi:10.1186/s12916-024-03590-x)
Supplement: Supplementary file 1 — Supplementary Material 1. [file 12916_2024_3590_MOESM1_ESM.pdf]

Unleashing the Full Potential of Digital Outcome Measures in Clinical Trials:
Eight Questions that Need Attention
Supplementary File

We display additional simulation results where participants are randomised with a 1:1 ratio to treatment (all other settings are the same as described in the main text of the paper).

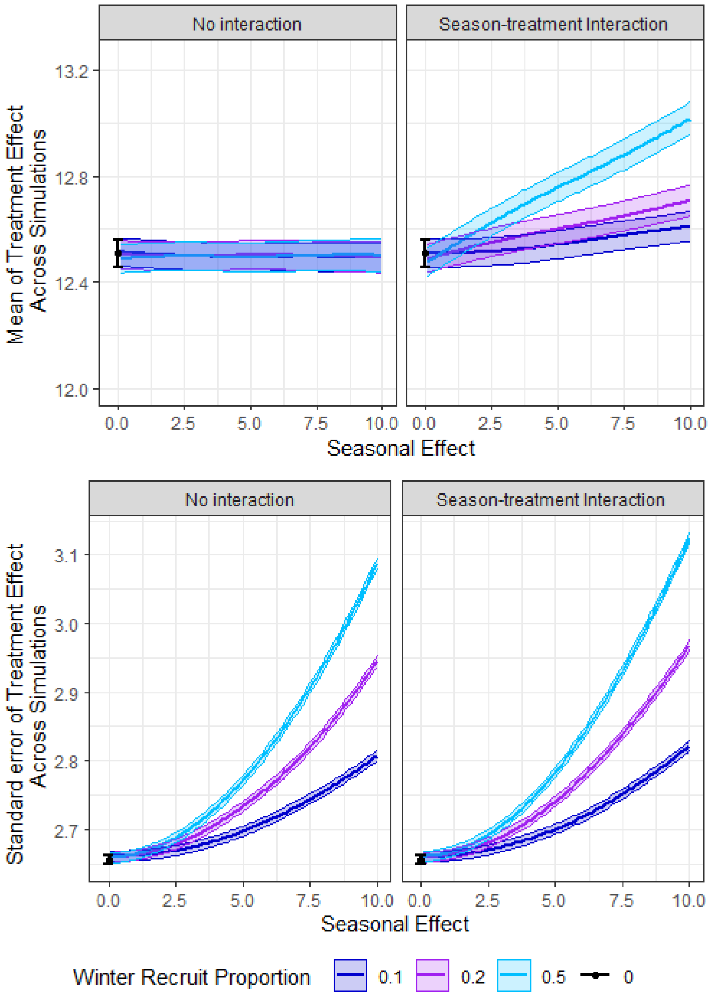

Figure 1: Seasonality. Plots show estimated mean of treatment effect (top) and its standard error (bottom) without interaction between season and treatment (left) and with an interaction (right). The seasonal effect varies between 0 and 10. Error bars indicate 1.96× Monte Carlo error. The black error bar indicates the scenario under no seasonal effect. Dark blue, purple and teal lines indicate that the proportion of patients recruited in winter are 0.1, 0.2 and 0.5, respectively. Results are based on 10,000 simulations.

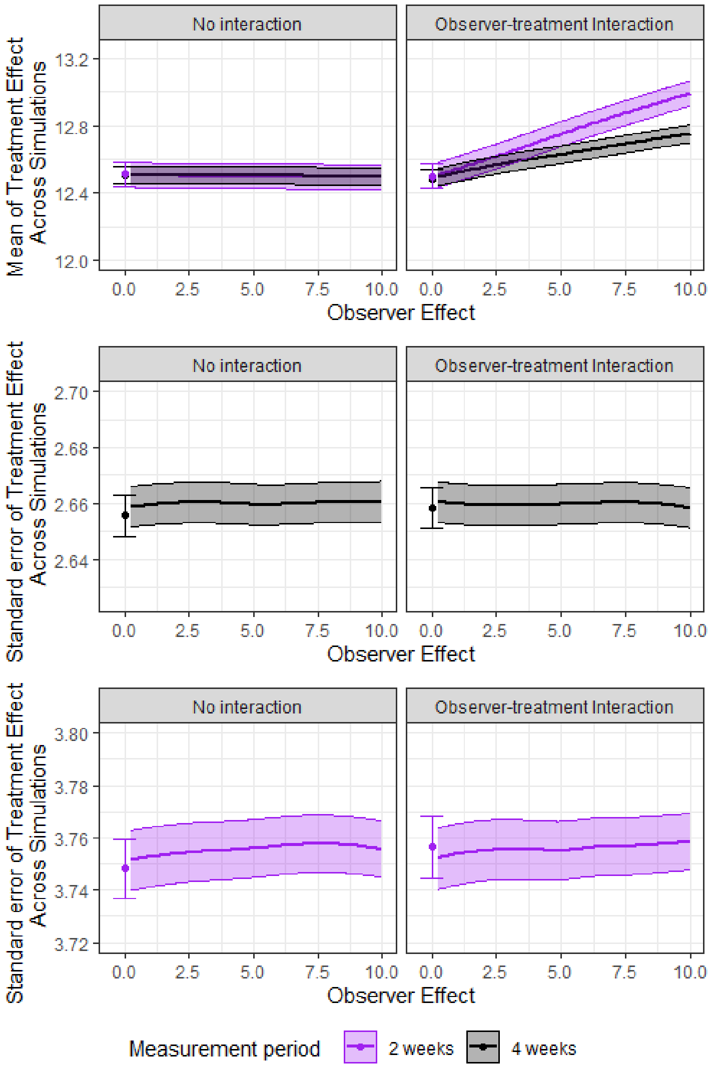

Figure 2: Observer Effect and Measurement period. Plots show the estimated mean of treatment effect (top), standard error when the measurement period is four weeks (middle) and standard error when the measurement period is two weeks (bottom), without an interaction between the observer effect and treatment (left) and with an interaction (right). Note that the scale of the y-axis is different for the middle and bottom panels. The observer effect varies between 0 and 10. Error bars indicate 1.96× Monte Carlo error. Grey lines indicate when the measurement period is four weeks, and purple lines indicate when the measurement period is two weeks. Results are based on 10,000 simulations.

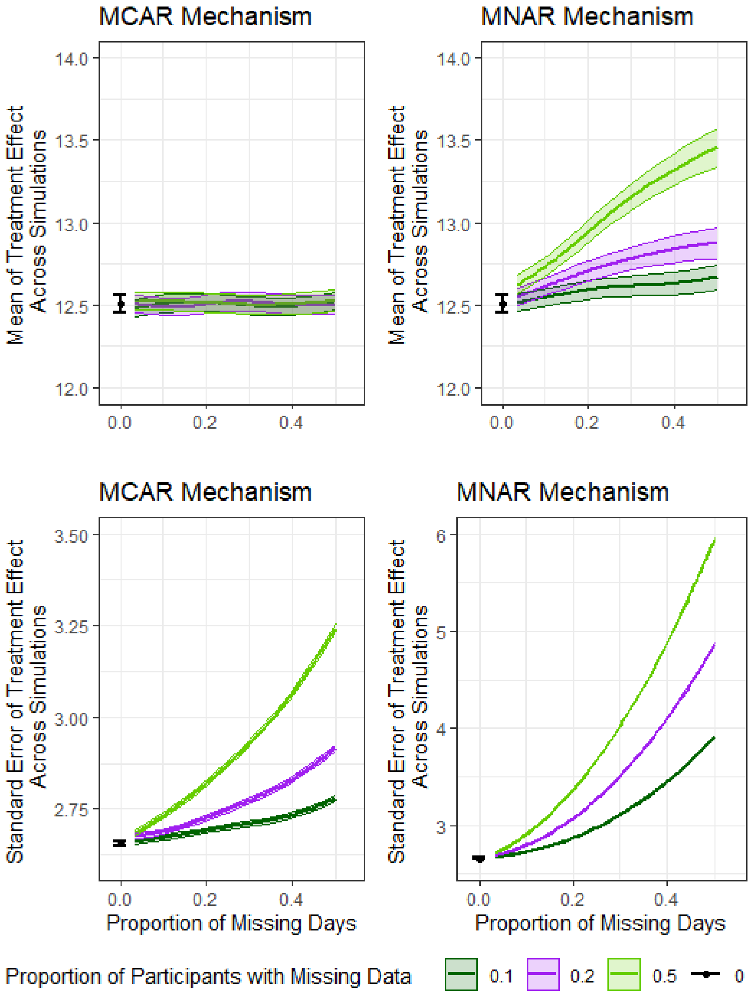

Figure 3: Missing Data. Plots show the change in the estimated mean of treatment effect (top) and its standard error (bottom) when data are MCAR (left) and MNAR (right). The proportion of days that are missing completely at random varies between 0.05 and 0.5. Error bars indicate 1.96× Monte Carlo error. The black error bar indicates the scenario under complete data. Dark green, purple and light green lines indicate that the proportion of patients with missing data are 0.1, 0.2 and 0.5, respectively. Note that the scale of the y-axis is different for the left and right panels for standard error. Results are based on 10,000 simulations.
